# Characteristics and Surgical Outcomes for Primary Malignant Melanoma of the Esophagus

**DOI:** 10.1038/srep23804

**Published:** 2016-04-01

**Authors:** Shugeng Gao, Jiagen Li, Xiaoli Feng, Susheng Shi, Jie He

**Affiliations:** 1 Department of Thoracic Surgery, Cancer Institute and Hospital, Chinese Academy of Medical Sciences and Peking Union Medical College, Panjiayuannanli No. 17, Chaoyang District, Beijing, 100021, The People’s Republic of China; 2 Department of Pathology, Cancer Institute and Hospital, Chinese Academy of Medical Sciences and Peking Union Medical College, Panjiayuannanli No. 17, Chaoyang District, Beijing, 100021, The People’s Republic of China

## Abstract

Primary malignant melanoma of the esophagus (PMME) is an extremely rare disease with poor prognosis. We summarized and analyzed the characteristics of 17 PMME patients (with average age of 57.5 ± 10.3 years) who had received surgical resection in our center. The majority (13/17, 76.5%) of the patients were male. The percentage of patients with smoking and alcohol consumption was 41.2% and 23.5%, respectively. The preoperative diagnosis rate was 35.3%. Lymph node metastasis mainly involved the mid-lower mediastinal and upper abdominal area. Primary tumors that invaded beyond the submucosa layer (T2–T4) had much higher tendency of lymph node metastasis than those restricted to the submucosa layer (T1) (6/8, 75.0% vs. 3/9, 33.3%, *p* = 0.086). The 1-year and 5-year survival rate of the patients was 51% and 10%, respectively, with median survival time being 18.1 months. Survival analysis showed that TNM stage was a predictor for PMME prognosis (median survival time of 47.3 months vs. 8.0 months for stage I/II vs. stage III, respectively, *p* = 0.018), and multivariable Cox regression analysis revealed the independence of its prognostic value [HR (95% CI): 5.678 (1.125–28.658), *p* = 0.035].

Primary malignant melanoma of the esophagus (PMME) is an extraordinarily rare disease which comprises only 0.1–0.2% of all tumors of esophagus^1^,^2^. PMME is a highly aggressive malignancy with high potential of metastasis^3^. Although this disease has been reported by Pava in as early as 1963^3^, its prognosis remains dismal with the 5-year survival rate of 4% to 37%^1^,^4^,^5^. A comprehensive treatment strategy has not been established due to lack of enough cases and strong evidence. Until recently, the mainstay for the treatment of PMME is still radical resection of the tumor. The roles of adjuvant and neoadjuvant therapy remained unclear for lack of evidence. For extremely rare occurrence, the number of documented cases has been very limited. By the year 2011, only 337 cases had been reported worldwide, most of which being single case reports^2^. Better understanding of the clinical and pathological features of the disease will help to improve the effectiveness of diagnosis and treatment, which calls for larger scale case summary. Up to now, the largest cohort of PMME was presented by Wang *et al.* in 2013, which included 13 cases. In the present study, we summarized the clinical and pathological characteristics of 17 PMME patients who had received esophagectomy in Cancer Institute and Hospital, Chinese Academy of Medical Sciences and Peking Union Medical College, among whom the earliest case was treated and diagnosed in 1975^6^. We also evaluated the prognosis related factors of the patients. To our knowledge, this is the largest series of PMME patients in the world.

## Results

### Demographic and clinical characteristics

The demographic and clinical presentations of the 17 PMME patients had been shown in Table 1. In summary, the mean age was 57.5 ± 10.3 years, ranging from 41 to 73. The majority (76.5%, 13/17) of the patients were male, and the percentage of tobacco and alcohol use was 41.2% (7/17) and 23.5% (4/17), respectively. A smoker was defined as consumption of ≥1 pack/day cigarette for ≥6 months. Alcohol consumption was calculated in terms of ml of ethanol consumed daily, where 355 ml of beer, 118 ml of wine, or 44 ml of hard liquor each was considered to be equivalent to 12.0 g of ethanol. Two of the patients had family history of upper gastrointestinal tract (UGI) cancers. However, none of them had family history of malignant melanoma. Of the 17 patients, 15 (88.2%) presented with dysphagia, which is the most common chief complaint of PMME patients^3^. The other 2 patients presented with haematemesis or bellyache, respectively. The average time interval between the onset of symptom and diagnosis was 2.9 ± 2.7 months (range: 0.1 to 12). One patient had comorbidity of hypertension and diabetes, and another had coronary heart disease.

**Figure 1:**
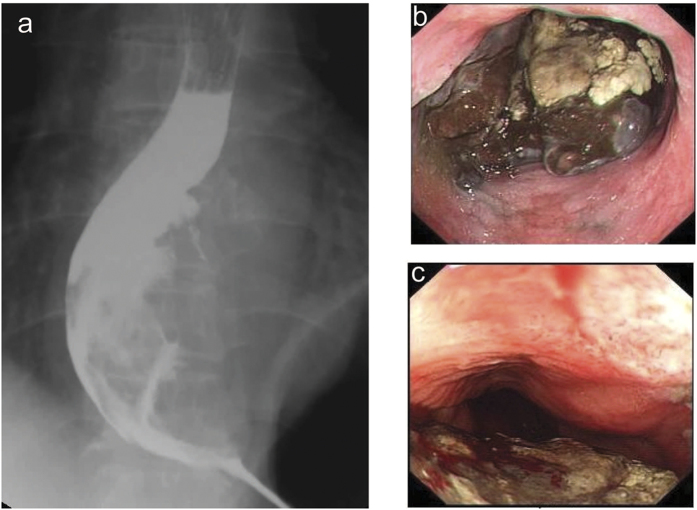
Barium swallow exam revealed an irregular filling defect on the lower third of the esophagus, causing mucosa destruction (**a**). Upper gastrointestinal endoscopy highlighted an irregular mass in the esophageal lumen with rough and pigmented surface (**b**) which was easily bleeding (**c**).

**Figure 2:**
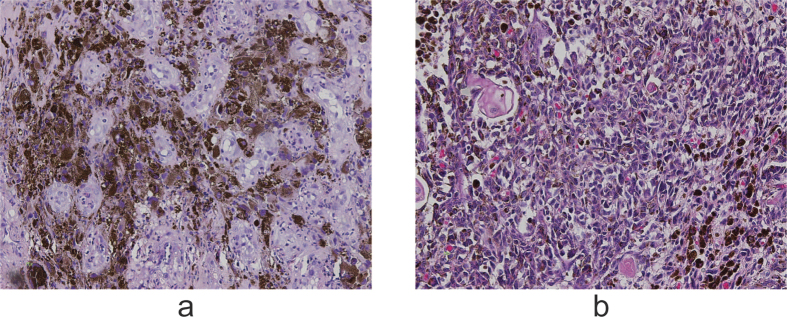
Immunohistochemical staining with HMB45 (human melanoma black 45) antibody revealed positive tumor cells in the esophageal mucosa of preoperative endoscopic biopsy (**a**, ×200). H&E staining identified malignant melanoma cells in the lamina propria of the esophagus (**b**, ×200)

All the patients took barium swallow and endoscopic examination before surgery. For most of them, barium swallow revealed mucosa destruction, irregular filling defect, and narrowness of the esophageal lumen (Fig. 1a). Irregular mass with rough, eroded, friable and easily bleeding surface was the common finding in endoscopic examination (Fig. 1b,c). In 7 (41.2%) of the patients, the esophageal mass was surrounded with a pigmented surface, which helped the diagnosis before surgery. Among the 17 patients, 6 (35.3%) were accurately diagnosed as malignant melanoma of esophagus before surgery by endoscopic biopsy (Fig. 2a). In those without accurate preoperative diagnosis, 3 (27.3%) were subjected to surgery as squamous cell carcinoma and one (9.1%) as adenocarcinoma while the other 6 (54.5%) were only roughly diagnosed as “poorly differentiated carcinoma”. Only 1 out of the 17 patients had preoperative radio- therapy (radiation at the dose of 45 Gy for 23 days). In addition, serum tumor markers including carcinoembryonic antigen (CEA), cyfra21-1 (cytokeratin 19 fragment), squamous cell carcinoma (SCC), and CA72-4 (tumor-associated glycoprotein 72) were tested in 12 of the patients preoperatively, yet none presented positive results. Comprehensive metabolic panel were also tested for each patient, yet none was found to have specific relationship with the disease.

### Surgical and pathological features

Subtotal esophagectomy and esophagogastrostomy plus systemic mediastinal and abdominal lymph node dissection were performed for all the patients. The average time of surgery was 191.8 ± 34.5 minutes with blood loss of 311.8 ± 153.6 ml (Table 2). One patient (5.9%) died perioperatively (57^th^ day after surgery) for respiratory failure and severe sepsis caused by anastomotic leak and subsequent bronchopleural fistula. Except for this patient, no leakage occurred in others. Besides, arrhythmia occurred in 6 (35.3%) patients (including the patient with leakage, Table 2). Thus the overall complication rate was 35.3%.

As shown in Table 2, the average diameter of the tumors was 5.9 ± 2.3 cm. The majority (16/17, 94.1%) of the tumors were located in the middle [7 (41.2%)] or lower [9 (52.9%)] part of the esophagus. The tumor was found at the upper esophagus in only 1 patient (5.9%, 1/17), which was consistent with other reports^3^,^7^.

Pathological examination (Fig. 2b) revealed that the lesion was restricted to the mucosa (T1a) in 1 patient (5.9%); whereas 8 (47.0%) patients had tumor invasion to the submucosal layer (T1b). The number of patients with tumor extension to the deep muscular layer (T2), fibrous membrane (T3) and beyond the serosa (T4a) was 1 (5.9%), 3 (17.6%), and 4 (23.5%), respectively.

The resected specimens were subjected to immunohistochemical examination and the positive rates were: HMB-45 (15/16), Melan-A (15/16), S100 (14/16), vimetin (8/13), Ki-67 (4/9), AE1/AE3 (1/9), CD117 (1/2) and bcl-2 (1/2).

The number of lymph node dissected in surgery was 17.2 ± 9.4, averagely. As to N stage, 8 (47.0%) patients was found to have no lymph node metastasis (N0), and the number of patients having 1–2 (N1), 3–6 (N2), and >7 (N3) metastatic lymph nodes was 6 (35.3%), 0, and 3 (17.6%), respectively (Table 2). Up to down, the lymph node area of esophageal cancer could be divided into 4 groups: the cervical, upper mediastinal, mid-lower mediastinal, and the upper abdominal group^8^,^9^,^10^. It was observed that for all the 9 patients with metastatic lymph nodes, lesions involved lymph nodes of the mid-lower mediastinal and upper abdominal groups, regardless of the location of primary tumors. Furthermore, for both mid third and lower third esophagus PMME, node metastasis was likely to occur at the upper abdominal area (2/3 and 5/6 for mid and lower esophagus, respectively). Moreover, comparison of the invasion depth of primary tumors and lymph node metastases revealed that tumors restricted to the submucosa layer (T1) were less likely to have lymph node metastasis (3/9, 33.3%) than those with deeper invasion (T2–T4) (6/8 75.0%). Although the difference was not statistically significant, yet the *p* value (0.086) was marginal, probably owning to limited sample size.

### Survival factors for PMME patients

**Figure 3:**
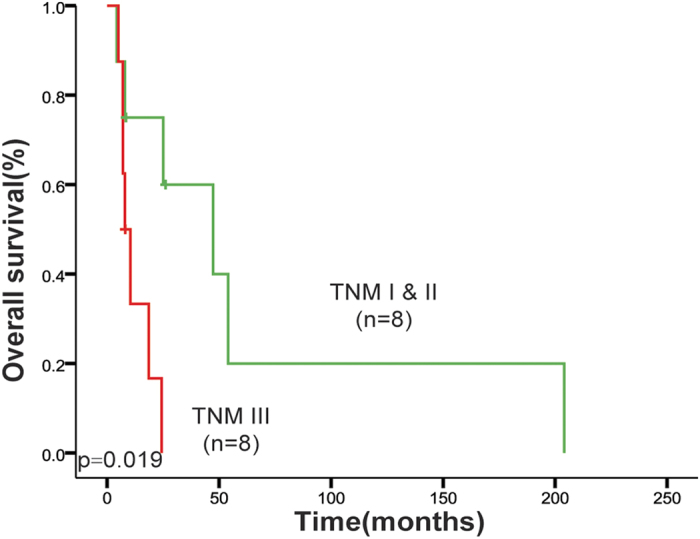
TNM stage was associated with overall survival of PMME patients. Kaplan-Meier survival curves of stage III vs. stage I~II patients. The p value was calculated by log-rank test.

The median survival time (MST) of the 17 patients was 18.1 months, with 1-year and 5-year survival rate being 51% and 10%, respectively. In order to analyze the relationship between the clinical and pathological factors and the overall survival (OS) of PMME patients, log-rank test was performed for each factor. The patient who died perioperatively for severe complication was excluded in the analysis. We found that TNM stage was significantly associated with the prognosis of PMME patients. Comparison revealed that TNM stage III patients had significantly shorter post-operative OS than stage I and II patients (MST 8.0 vs. 47.3 months, *p* = 0.0185, Fig. 3). Multivariable Cox regression analysis showed that TNM stage was an independent prognostic factor for PMME patients [HR (95% CI): 5.678 (1.125–28.658), *p* = 0.0355, Table 3]. The patients with lymph node metastasis tended to have shortened OS, even though the difference was not significant (MST 10.4 vs. 47.3 months, p = 0.1871, Fig. 4).

## Discussion

In the present study, we summarized the characteristics of 17 PMME patients and analyzed the lymph node metastasis patterns and survival associated factors. We found that tumors with deeper invasion involved lymph nodes more frequently and that TNM stage was significantly associated with the clinical outcome of PMME.

The distribution of age and gender (ratio of male to female 13:4) of our cohort was similar with esophageal squamous cell carcinoma (ESCC)^11^, which is the major form of esophageal malignancies in China. However, the ratio of patients with tobacco or alcohol use was different from that of ESCC. It was reported that the majority of ESCC patients had the history of smoking and drinking^12^,^13^ so that tobacco and alcohol were considered as major risk factors of ESCC^12^,^14^. Our study showed that more than half of the PMME patients were non-users of tobacco and alcohol. Although it is inappropriate to conclude that the etiology and epidemiology of PMME is different cause the sample size is small, yet our finding suggests that this issue merits further investigation through a larger sample size.

The diagnosis of PMME for our patients was made depending upon post-operative pathological examination with immunohistochemical checkup. The majority of the specimens had positive expression of the classic immunomarkers, like MB-45, Melan-A, S100, and vimetin, which is consistent with other reports^6^,^10^,^15^,^16^. However, accurate diagnosis before surgery is difficult even with endoscopic biopsy. When tumors lack the dark surface grossly and the melanin granules microscopically, they could be easily confounded with poorly differentiated carcinomas. Additionally, our results showed that none of the traditional serum tumor markers or biochemical index was elevated in the PMME patients, indicating the lack of serum biomarkers for early detection and recurrence surveillance for the disease, which further leads to its dismal prognosis.

Wang and colleagues summarized 13 PMME patients and reported that PMME located at the lower third of the esophagus, lymph node metastasis tended to occur at the upper mediastinal area, which was similar to ESCC^10^,^17^. However, our patients has shown a different pattern. Upper abdominal was the area where node metastasis most frequently occurred. None of the 9 patients with lymph node metastasis involved the upper mediastinal group. This discrepancy indicates that observations and summaries of a larger sample size are guaranteed to reveal the lymph node metastasis pattern for PMME.

The PMME has poor prognosis, with 5-year survival rate ranging from 4% to 37%^1^,^4^,^5^. Our study presented similar results, with 5-year survival rate being 10% and median survival time of 18.1 months. The only patient who had survived over 5 years died 17 years after surgery. However, she died of lung cancer rather than the progress of PMME^6^. This is a TNM stage I (T1aN0M0) female patient and it has been noticed that this patient was also the only one who had received neoadjuvant radiotherapy (45 Gy) before surgery. It could not be evaluated at present whether or how the neoadjuvant radiotherapy had contributed to her longer survival. Neither could we evaluate its role in the treatment of PMME patients for lack of data of similar patients. However, our data indicates the value of further study on the role of neoadjuvant radiotherapy in the treatment of PMME.

As to the survival predictor for PMME patients, we have found the prognostic value of TNM stage. This indicates that early detection of the disease is critical for better survival. Previous studies on both Japanese and Chinese patients have shown that node metastasis was an independent prognostic factor^10^,^18^. Our study also showed that lymph node positive patients did tend to have poor survival. The difference was not significant, probably due to a limited sample size. Additionally, our results revealed that primary tumor penetrating beyond the submucosa layer was more likely to metastasize to lymph nodes, indicating the importance of early discovery and treatment of PMME for the better outcome of the patients. With the absence of serum indicators, early detection should depend on barium swallow, CT or MRI, and more importantly, endoscopic examination.

In this study, all the 17 patients enrolled received surgical resection. However, the complete and accurate information of the adjuvant therapy they received after surgery was not available. Thus our study has limitations in that how adjuvant chemo- or radio-therapy may affect the clinical outcome was not analyzed. Although quite a few reports have stated that adjuvant therapy plays a palliative role^3^,^7^, more cases with complete and accurate information are needed for valid conclusion. Besides, as the follow up information of 3 patients was incomplete and their follow up time were much shorter than 5 years, there might be bias in the 5-year survival rate and the median survival time. Also, even though this is the largest group of PMME patients ever reported, yet the sample size is still too small to provide enough power for most of the analysis, especially for survival comparisons. Thus accumulation of more cases is the key to better understanding of the disease.

## Conclusion

The clinical and pathological features of 17 PMME patients were summarized and the lymph node metastasis pattern and survival associated factors were also evaluated. Early detection of the disease and radical resection of the tumor are critical for better survival of the PMME patients.

## Materials and Methods

### Patients and clinical data

After searching and reviewing medical records database, all patients (17 totally) diagnosed as primary malignant melanoma of the esophagus (PMME) who had received surgical resection in the Cancer Institute and Hospital, Chinese Academy of Medical Sciences (CAMS) between 1975 and 2015 were collected and enrolled in the study^6^. The demographic, clinical, and pathological characteristics of these patients were obtained through retrospective review of their medical records, which included age, gender, tobacco and alcohol use, family history, symptoms, endoscopic and radiographic examination, serum tumor-marker levels, tumor size, location, TNM stage, comorbidities and complications. The tumor histology was reviewed and confirmed by two independent pathologists (F-XL and S-SS). TNM stage of the patient was based on the American Joint Committee on Cancer staging manual (7th edition). Follow-up of the patients was obtained by telephone, mail, and personal interview. The median follow-up time was 8.4 months.

This study has been approved by Institutional Review Board (IRB) committee at Cancer Institute and Hospital, CAMS. Informed consent was obtained from each of the participant before the operation. All procedures were conducted according to the guidelines approved by the ethics committee at Cancer Institute and Hospital, CAMS.

### Statistical analysis

Data were presented as mean values plus standard deviations unless otherwise stated. For comparison of categorical variables, chi square test was used. Log-rank test was applied to analyze the correlation between clinicopathological factors and the survival of PMME patients. Univariate and multivariate Cox regression analyses using stepwise approach were performed to identify independent prognostic factors for PMME patients. Selected co-variables included age, gender, tobacco use, alcohol use, tumor location, T stage, lymph node metastasis, TNM stage, and perioperative complication. A p value of <0.05 was considered statistically significant.

## Additional Information

**How to cite this article**: Gao, S. *et al.* Characteristics and Surgical Outcomes for Primary Malignant Melanoma of the Esophagus. *Sci. Rep.* **6**, 23804; doi: 10.1038/srep23804 (2016).

## Figures and Tables

**Figure 4:**
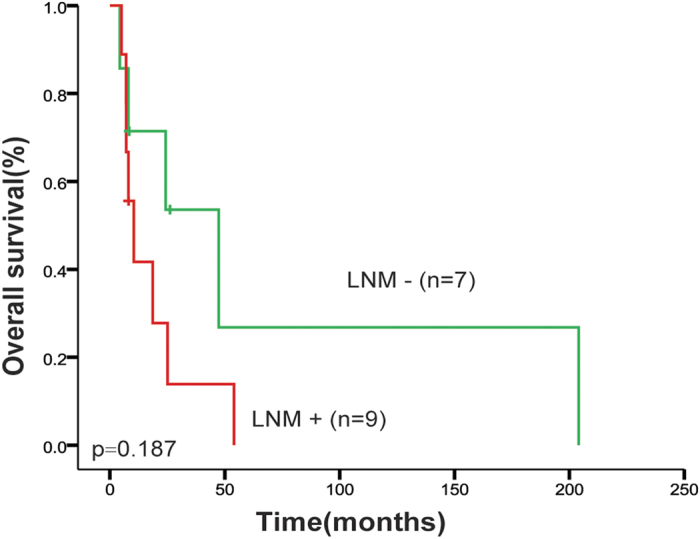
Association of lymph node metastasis and survival of PMME patients. Kaplan-Meier curves of node metastasis involvement status. LNM: lymph node metastasis.

**Table 1:** Demographic and clinical characteristics of the PMME patients.

Case No.	Age	Gender	Tobacco use	Alcohol use	Family history^a^	Chief complaint	Months between onset and diagnosis	Preoperative diagnosis	Preoperative treatment	Comorbidity	Vital status	Survival time^b^
1	73	male	no	no	no	dysphagia	3.0	ESCC	no	no	Dead	7.1
2	68	male	yes	no	yes	dysphagia	12.0	ESCC	no	no	Alive	26.1
3	71	male	no	no	yes	dysphagia	2.0	MME	no	no	Alive	8.1
4	54	male	yes	yes	no	dysphagia	2.0	MME	no	no	Alive	8.4
5	63	male	yes	no	no	dysphagia	2.0	MME	no	no	Dead	25.1
6	67	female	no	no	no	dysphagia	2.0	MME	no	no	Dead	47.3
7	43	female	no	no	no	dysphagia	0.7	MME	no	no	Dead	4.3
8	55	male	no	no	no	dysphagia	1.0	Unspecified	no	no	Dead	1.9
9	60	male	no	no	no	dysphagia	1.7	Unspecified	no	no	Dead	18.6
10	70	male	yes	yes	no	dysphagia	6.0	Unspecified	no	HTN + DM	Dead	10.4
11	50	male	yes	no	no	haematemesis	1.3	EAC	no	no	Dead	24.3
12	57	male	no	no	no	dysphagia	4.0	Unspecified	no	no	Dead	7.0
13	43	male	yes	yes	no	dysphagia	4.0	ESCC	no	no	Dead	8.0
14	56	male	no	no	no	dysphagia	3.0	MME	no	no	Dead	54.0
15	41	female	no	no	no	belly ache	1.0	Unspecified	no	no	Dead	5.0
16	46	female	no	no	no	dysphagia	3.0	Unspecified	Radiotherapy	CHD	Dead	204
17	60	male	yes	yes	no	dysphagia	2.0	ESCC	no	no	Dead	8.0

^a^Family history of UGI(upper gastro-intestinal tract) cancer.

^b^months. ESCC, esophageal squamous cell carcinoma. MME, malignant melanoma of esophagus. EAC, esophageal adenocarcinoma. HTN, hypertension. DM, diabetes mellitus. CHD, coronary heart disease.

**Table 2:** Surgery and pathological features of the PMME patients.

Case No.	Surgery time (min)	Blood loss (ml)	Complication	Tumor size (cm)	Location	Invasion depth	T stage	Dissected LN	LNM	N stage	TNM stage	LNM area
1	210	600	Arrhythmia	8.0	Mid	Fibrous membrane	3	20	2	1	III	Upper abdominal
2	190	200	Arrhythmia	7.5	Mid	Fibrous membrane	3	19	0	0	II	–
3	220	100	Arrhythmia	7.0	Mid	Submucosa	1b	43	9	3	III	Mid-lower mediastinal
4	170	100	No	4.2	Lower	Submucosa	1b	19	0	0	I	–
5	200	200	Arrhythmia	4.0	Mid	Submucosa	1b	22	1	1	II	Upper abdominal
6	200	200	Arrhythmia	2.7	Upper	Submucosa	1b	14	0	0	I	–
7	200	200	No	4.5	Mid	Submucosa	1b	20	0	0	I	–
8	170	300	Arrhythmia + Leakage + BPF	5.0	Mid	Submucosa	1b	21	0	0	I	–
9	210	300	No	6.0	Lower	Beyond serosa	4a	23	1	1	III	Upper abdominal
10	170	200	No	6.5	Lower	Deep muscular	2	19	11	3	III	Mid-lower mediastinal
11	130	400	No	2.5	Lower	Beyond serosa	4a	11	0	0	III	–
12	210	600	No	7.0	Lower	Beyond serosa	4a	2	2	1	III	Upper abdominal
13	270	400	No	10.0	Mid	Submucosa	1b	16	0	0	I	–
14	210	500	No	5.0	Lower	Submucosa	1b	9	1	1	II	Upper abdominal
15	120	400	No	3.0	Lower	Beyond serosa	4a	8	7	3	III	Upper abdominal
16	180	300	No	10.0	Lower	Mucosa	1a	3	0	0	I	–
17	200	300	No	7.0	Lower	Fibrous membrane	3	23	1	1	III	–

BPF, bronchopleural fistula. LN, lymph node. LNM, lymph node metastasis. TNM, tumor, lymph node, metastasis.

**Table 3:** Univariate and multivariate Cox regression analyses of the clinical and pathological factors with survival of PMME patients (n = 16).

		Univariate analysis	Multivariate analysis	Hazard ratio (95% CI)	*P*
		Hazard ratio (95% CI)	*P*		
Age	>60 vs. ≤60	0.717 (0.208–2.479)	0.5995	−	−
Gender	Female vs. Male	0.783 (0.196–3.126)	0.7288	−	−
Tobacco use	Yes vs. No	1.026 (0.294–3.574)	0.9684	−	−
Alcohol use	Yes vs. No	2.051 (0.448–9.397)	0.3550	−	−
Tumor location	Lower vs. Upper + Middle	0.927 (0.280–3.070)	0.9013	−	−
T	T2 + T3 + T4 vs. T1	3.262 (0.818–13.004)	0.0938	−	−
N	N1 + N2 + N3 vs. N0	2.211 (0.655–7.469)	0.2013	−	−
TNM	III vs. I + II	5.678 (1.125–28.658)	0.0355	5.678 (1.125–28.658)	0·0355
Complication	Yes vs. No	0.577 (0.150–2.216)	0.4231	−	−

CI, confidential interval.
